# Supplementary material for: Clinical Outcomes in Fibrolamellar Hepatocellular Carcinoma Treated with Immune Checkpoint Inhibitors
Source: Cancers (Basel). 2022 Oct 30;14(21):5347. doi: 10.3390/cancers14215347 (PMC9655068; doi:10.3390/cancers14215347)
Supplement: Supplementary file 1 [file cancers-14-05347-s001.zip › cancers-1961765-supplementary.pdf]

Supplemental Figures

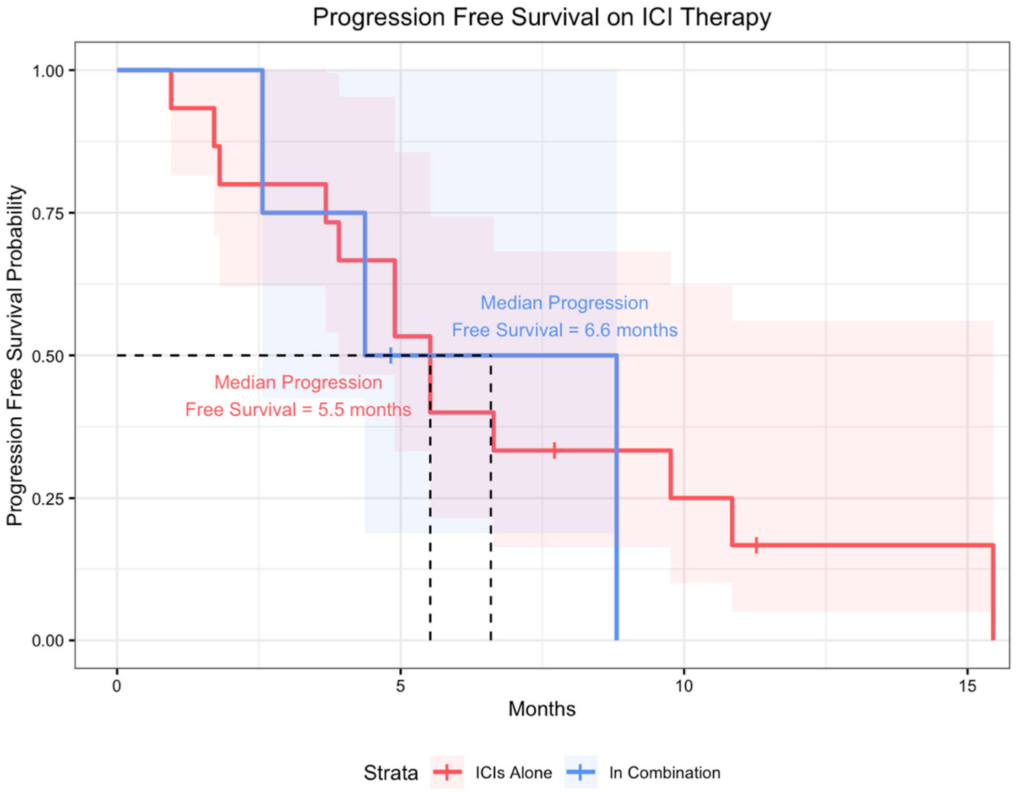

Figure S1. Progression free survival of 19 patients with FLC treated with ICI disaggregated by presence of combinatory treatment regimen. Of 19 patients, 15 received ICIs alone and 4 received ICIs in combination with other therapies. The shaded regions represent the 95% confidence intervals.

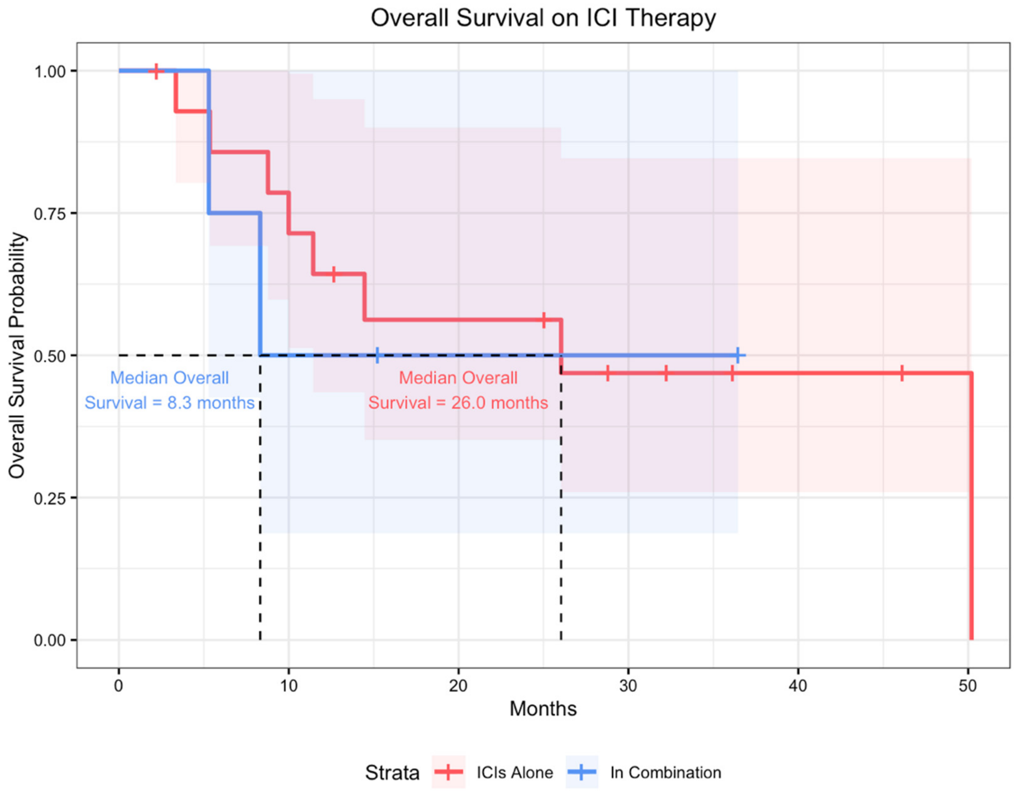

Figure S2. Overall survival of 19 patients with FLC treated with ICI therapy disaggregated by presence of combinatory treatment regimen. Of 19 patients, 15 received ICIs alone and 4 received ICIs in combination with other therapies. The shaded regions represent the 95% confidence intervals.

Table S1. Adverse events in FLC patients due to ICI therapy, disaggregated by presence of combinatory treatment regimen.

**Number of patients experiencing any AE**	
Any ICI Regimen	10 (52.6%)
ICI in combination with non-ICI therapies	1 (25.0%)
**Number of patients who discontinued ICIs due to AE**	
Any ICI Regimen	2 (10.5%)
ICI in combination with non-ICI therapies	0 (0%)
**Any grade III or IV AE**	
Any ICI Regimen	4 (21.1%)
ICI in combination with non-ICI therapies	0 (0%)
**Average Number of AEs experienced by patients**	
Any ICI Regimen	1.1
ICI in combination with non-ICI therapies	0.25

**Specific AE Data** (Any ICI Regimen)	
Elevated LFTs	4 (21.1%)
Fatigue	4 (21.1%)
Hypothyroidism	2 (10.1%)
Nausea	2 (10.1%)
Anaphylaxis	1 (5.3%)
Diarrhea	1 (5.3%)
Hyperthyroidism	1 (5.3%)
Pneumonitis	1 (5.3%)
Pruritis	1 (5.3%)
Pyrexia	1 (5.3%)
Rash	1 (5.3%)
Vomiting	1 (5.3%)
